# Antibody landscapes of arboviral exposure across China revealed by high-throughput seroprofiling from a peptide epitope library

**DOI:** 10.1186/s40249-025-01399-1

**Published:** 2026-01-09

**Authors:** Nan Zhang, Wei Liu, Feng Zhu, Wan Ni Chia, Dai Kuang, Ying Luo, Yuxuan Han, Hua Pei, Lin-Fa Wang, Qianfeng Xia

**Affiliations:** 1 National Health Commission Key Laboratory of Tropical Disease Control, School of Life Sciences and Medical Technology, Hainan Medical University, Haikou, 571199 Hainan China; 2 Programme in Emerging Infectious Diseases, Duke-National University of Singapore Medical School, Singapore, Singapore; 3 Department of Immunology, University of Texas Southwestern Medical Center, Dallas, TX USA; 4 Chinese Academy of Sciences Key Laboratory of Pathogen Microbiology and Immunology, Institute of Microbiology, Chinese Academy of Sciences, Beijing, China; 5 The Second Affiliated Hospital and the Transplantation Institute, Hainan Medical University, Hainan, China

**Keywords:** Arboviruses, Serosurveillance, Phage immunoprecipitation sequencing, Regional differences

## Abstract

**Background:**

Arboviral infections impose significant public health challenges globally, yet routine surveillance typically captures only symptomatic infections, underestimating the true extent of exposure. Insights into how regional and demographic factors influence population immunity are essential for targeted surveillance and prevention, but such multidimensional insights remain limited. This study aimed to quantify population-level arboviral sero exposure and delineate the effects of regional and demographic factors on immunity to inform targeted surveillance and prevention.

**Methods:**

We utilized a programmable phage display platform, ArboScan, which evaluates antibody binding to overlapping peptides that represent the proteomes of 691 human and zoonotic arboviruses. We profiled baseline antibody reactivity in serum samples from 400 healthy individuals, collected before the dengue outbreaks reported in Hainan in 2019. Antibody reactivity was quantified as normalized fold-change (FC) values relative to negative controls, and analyzed by region, sex, and age. Normality was assessed using the Shapiro-Wilk test. Two-group comparisons were conducted using independent two-sample t tests for normally distributed data or Mann-Whitney U tests otherwise; comparisons among > 2 groups were performed using One-way Analysis of Variance for normally distributed data.

**Results:**

Regional ranking by mean product fold change (MPFC) showed northern enrichment for bluetongue virus (MPFC = 3.56), whereas southern cohorts were enriched for mosquito-borne arboviruses-dengue virus (MPFC = 3.54), *Alagoas vesiculovirus* (MPFC = 3.50), and *Venezuelan equine encephalitis virus* (MPFC = 3.39). Females exhibited higher FC than males for selected arboviral families (*P* < 0.001). By family-level analysis, *Flaviviridae*, *Togaviridae*, and *Phenuiviridae* showed no age-stratified differences (*P* > 0.05). High fold-change values were detected for non-arboviral viruses such as human cytomegaloviruses and human adenoviruses across all regions.

**Conclusions:**

Our findings reveal distinct regional and demographic patterns of arboviral antibody reactivity in China, reflecting differing histories of exposure and potentially informing region-specific surveillance strategies. The stable antibody levels across age groups, together with higher fold-change values in females, underscore the influence of biological and social factors on arboviral immunity. The ArboScan platform, and programmable peptide display platforms in general, offer a scalable approach to characterize population-level immunity and could enhance early detection and public health preparedness in arbovirus-endemic areas.

**Graphical Abstract:**

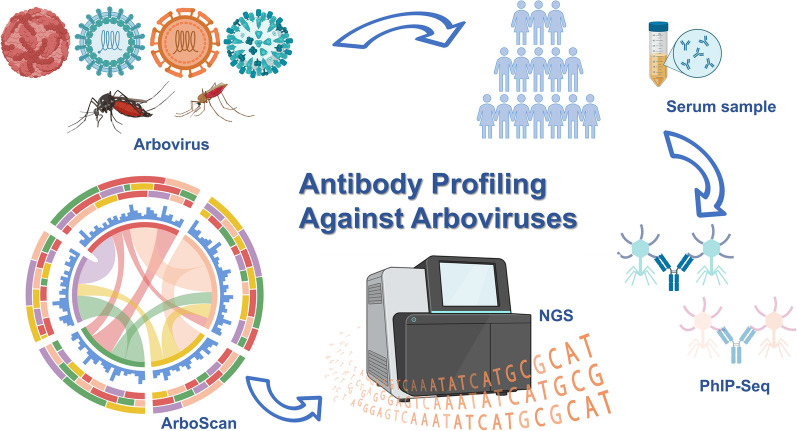

## Background

Vector-borne diseases, including arboviral infections, account for over 17% of all infectious diseases worldwide, posing a significant public health threat [1]. These include major pathogens such as dengue virus, Zika virus, and yellow fever virus, which infect millions of people annually and cause thousands of deaths worldwide [2, 3]. These vector-borne viruses span multiple families and exhibit substantial genetic diversity, complex transmission dynamics, and extensive serological cross-reactivity [4, 5].

Infectious diseases exhibit distinct epidemiological patterns across geographical regions due to variations in climate, environmental factors, and human activity [6]. However, large-scale serological studies in healthy populations remain scarce, especially in Asia, and in the few settings where such studies have been conducted, the results point to widespread silent exposure, for example, over 50% chikungunya virus IgG positivity in healthy adults in Sulawesi, Indonesia, and 85% arbovirus seropositivity in the Comoros [7, 8]. Recent India-focused syntheses further highlight persistent gaps in surveillance, diagnostics, and vector control, underscoring the need for scalable seroepidemiology to map baseline immunity as vectors expand into new areas [9].

Mapping the antibody reactome across diverse arboviruses not only reveals patterns of prior exposure, but also offers deeper insights into host immunity, antigenic imprinting, and potential cross-reactivity. Recent advances in high-throughput serological profiling have enabled unprecedented insights into the human antibody repertoire and its relationship to pathogen exposure. Among these, phage immunoprecipitation sequencing (PhIP-Seq) has emerged as a powerful platform that combines programmable phage display libraries, antibody immunoprecipitation, and next-generation sequencing to globally map antibody reactivity against hundreds of thousands of peptides [10]. Current phage display platforms have primarily focused on common arbovirus strains and lack the capability to characterize viral strain-specific antibody responses or map cross-reactivity between different arboviruses. To overcome these limitations, Morgenlander et al. developed ArboScan, a programmable phage display library containing 56-amino-acid peptides from the proteomes of 691 human and zoonotic arboviruses [11]. Additionally, its adaptability for both established and emerging arboviruses makes ArboScan an indispensable tool for large-scale arbovirus antibody profiling [12].

To address these limitations, we applied a high-throughput serological platform to systematically map antibody responses across a broad range of arboviruses. Using ArboScan, we profiled baseline antibody reactivity in serum samples from 400 healthy individuals in northern and southern China, collected before the dengue outbreaks reported in Hainan in 2019 [13]. Antibody responses were quantified as normalized fold-change (FC) values and analyzed by region, sex, and age. This multidimensional approach enables a more nuanced understanding of arboviral exposure and immune imprinting in the population, offering valuable insights for region-specific surveillance and immunization strategies.

## Methods

### Participant recruitment

Samples were selected using stratified sampling across northern (Beijing, Inner Mongolia, Heilongjiang, Jilin, Shandong, Gansu, Xinjiang, Shaanxi, Shanxi, Hebei, Liaoning, Henan) and southern (Hainan, Guangdong, Guangxi) China. All serum samples were collected before the dengue outbreaks reported in 2019 to minimize the likelihood of recent arboviral exposure and background cross reactivity. This regional stratification was chosen to capture potential differences in exposure history and ecological factors between northern and southern China. The northern and southern cohorts each comprised 200 asymptomatic adults (100 males, 100 females), with median ages of 51 (females) and 54 (males) years. The infant and pediatric groups were sampled exclusively in southern China due to recruitment constraints in the north. Participants were eligible if they had no documented history of arboviral infection, confirmed by serological testing and available medical records/test-requisition metadata. Exclusion criteria included recent travel to endemic areas, prior vaccination against any arthropod-borne virus, or occupational exposure to arbovirus-transmitting vectors or pathogens.

### ArboScan library generation

We utilized the ArboScan phage display library as previously described [10], which includes 25,138 unique protein sequences derived from 691 arbovirus species listed in GenBank as of 2017, along with 5 control viruses. Viral proteomes were selected based on literature review and taxonomy databases, including ArboCat and the International Committee on Taxonomy of Viruses, focusing on genera with evidence of arthropod-borne transmission. The proteomes of these potential arboviruses were meticulously identified through GenBank searches targeting specific viral genera or taxonomies, including *Coltivirus, Nyavirus, Flavivirus, Alphavirus, Nairovirus*, and many others. From these proteomes, unique polypeptide sequences were segmented into 56 amino acid peptides with overlapping sequences of 28 amino acids, with duplications removed. Peptides that differed by fewer than three amino acids were further refined or excluded, particularly for a subset that included 11 viruses exclusively infecting arthropods and 7 viruses considered of lower priority for human health. Oligonucleotides encoding these peptides were synthesized by Twist Bioscience (South San Francisco, California, USA) and subsequently cloned into a mid-copy T7 bacteriophage display system, following established protocols [14]. The preparation of phage libraries was conducted according to previously described methods, ensuring the integrity and functionality of the library for comprehensive viral interaction studies [14].

### Phage immunoprecipitation sequencing (PhIP-Seq)

We applied PhIP-Seq technology to characterize serum antibody binding profiles against a comprehensive library of phage-displayed viral peptides, the ArboScan peptide library [11]. PhIP-Seq was performed following protocols as previously described [14]. In brief, 1 µl of serum from each participant was incubated overnight at 4 °C with the ArboScan phage library, which presents overlapping 56-amino-acid peptides from arboviral proteomes on the surface of T7 bacteriophages, at a coverage of approximately 1 × 10^10^ phage particles per library member to ensure sufficient representation of each peptide. Antibody-phage complexes were isolated using magnetic beads coated with protein A and G to capture IgG-bound virions. Following immunoprecipitation, bound phage DNA was extracted and amplified by PCR with indexed primers for sample barcoding. The resulting amplicons were pooled and sequenced on an Illumina HiSeq 4000 system (Illumina, San Diego, CA, USA) to quantify peptide-specific antibody binding. Negative control reactions lacking serum were included in parallel to assess background binding and normalize downstream fold change calculations.

### Fold change calculation and definition of antibody reactivity

To ensure the specificity and reliability of PhIP-Seq results, rigorous quality control measures were implemented. Each serum sample was tested in a single replicate. To monitor background signal and non-specific binding, negative controls–including buffer-only and no-serum reactions–were included in every PhIP-Seq run. To identify significantly reactive peptides, Tukey’s fences method was applied. For each peptide tile, the upper threshold was defined as Q3 plus 1.5 times the interquartile range (IQR), based on the distribution of read counts from negative control runs. Peptide-level antibody reactivity was quantified by calculating FC scores from normalized reads from each peptide from testing samples divided by the Tukey’s fences (Q3 + 1.5 × IQR) of the same peptide from the negative control runs. For a specific protein, mean product fold change (MPFC) was calculated by the mean FC across all peptides; mean per peptide flag (MPPF) was calculated as the mean positive rates of the peptides in the library.

### Statistics analysis

The Shapiro-Wilk test was used to assess the normality of fold change distributions. If the distributions were skewed, log transformation was applied to improve normality. For data that remained non-normally distributed, the Mann-Whitney U test was used to compare FC values. One-way Analysis of Variance (ANOVA) was applied for comparisons involving more than two groups, assuming approximate normality. Independent *t*-tests were used when comparing two normally distributed groups. To ensure data integrity, blinded analysis was conducted, keeping both laboratory personnel and statisticians unaware of sample identities to prevent analyst bias. All analyses were conducted in R 4.3.1 (R Foundation for Statistical Computing, Vienna, Austria) using the tidyverse, ggplot2, and stats packages. Significance was defined as *P* < 0.05 unless otherwise stated. All figures were generated using customized ggplot-based pipelines, and plots were color-coded by viral taxonomy or reactivity flag.

## Results

### Regional ranking of top arboviral signals

In this study, we utilized the ArboScan and profiled arboviral antibody responses in 400 healthy individuals from northern (*n* = 200) and southern (*n* = 200) China. Immune responses were quantified using normalized FC values relative to negative controls, enabling the identification of significant antibody reactivity. To further investigate regional variation in immune recognition, we ranked the top five viral species with the highest MPFC values in each cohort (Table 1). In northern China, the highest-ranking arbovirus was bluetongue virus (MPFC = 3.56). In contrast, southern China exhibited a distinct immune profile, with higher MPFC values observed against dengue virus (MPFC = 3.54), *Alagoas vesiculovirus* (MPFC = 3.50), and *Venezuelan equine encephalitis virus* (MPFC = 3.39).

**Table 1:** The top-5 viruses for the northern vs southern China cohort

Location	Order	Family	Genus	Species	MPFC	MPPF
N	*Caudovirales*	*Myoviridae*	NA	Stenotrophomonas phage vB_SmaS-DLP_6^†^	3.5592	1.0000
N	*Herpesvirales*	*Herpesviridae*	Cytomegalovirus	*Cercopithecine betaherpesvirus 5*^†^	4.1661	0.5152
N	*Picornavirales*	*Picornaviridae*	Enterovirus	*Rhinovirus A*^†^	3.7682	0.1544
N	NA	*Adenoviridae*	NA	*Pan troglodytes adenovirus*^†^	3.8143	1.0000
N	NA	*Reoviridae*	*Orbivirus*	*Bluetongue virus*	3.5607	1.0000
S	*Herpesvirales*	*Herpesviridae*	Cytomegalovirus	*Cercopithecine betaherpesvirus 5*^†^	3.5190	0.5197
S	*Mononegavirales*	*Rhabdoviridae*	*Vesiculovirus*	*Alagoas vesiculovirus*	3.4991	1.0000
S	NA	*Adenoviridae*	NA	Pan troglodytes adenovirus^†^	3.6388	1.0000
S	NA	*Adenoviridae*	*Flavivirus*	dengue virus	3.5418	0.5000
S	NA	*Togaviridae*	*Alphavirus*	*Venezuelan equine encephalitis virus*	3.3929	0.5000

N North, S South, NA not annotated, MPFC Mean Product Fold Change, MPPF Mean Per Peptide Flag

^†^non-arboviruses included for completeness and excluded from arboviral analyses (e.g., bacteriophages or human/animal viruses that are not vector-borne)

### Overall viral seroreactivity stratified by age, sex, and region

**Fig. 1:**
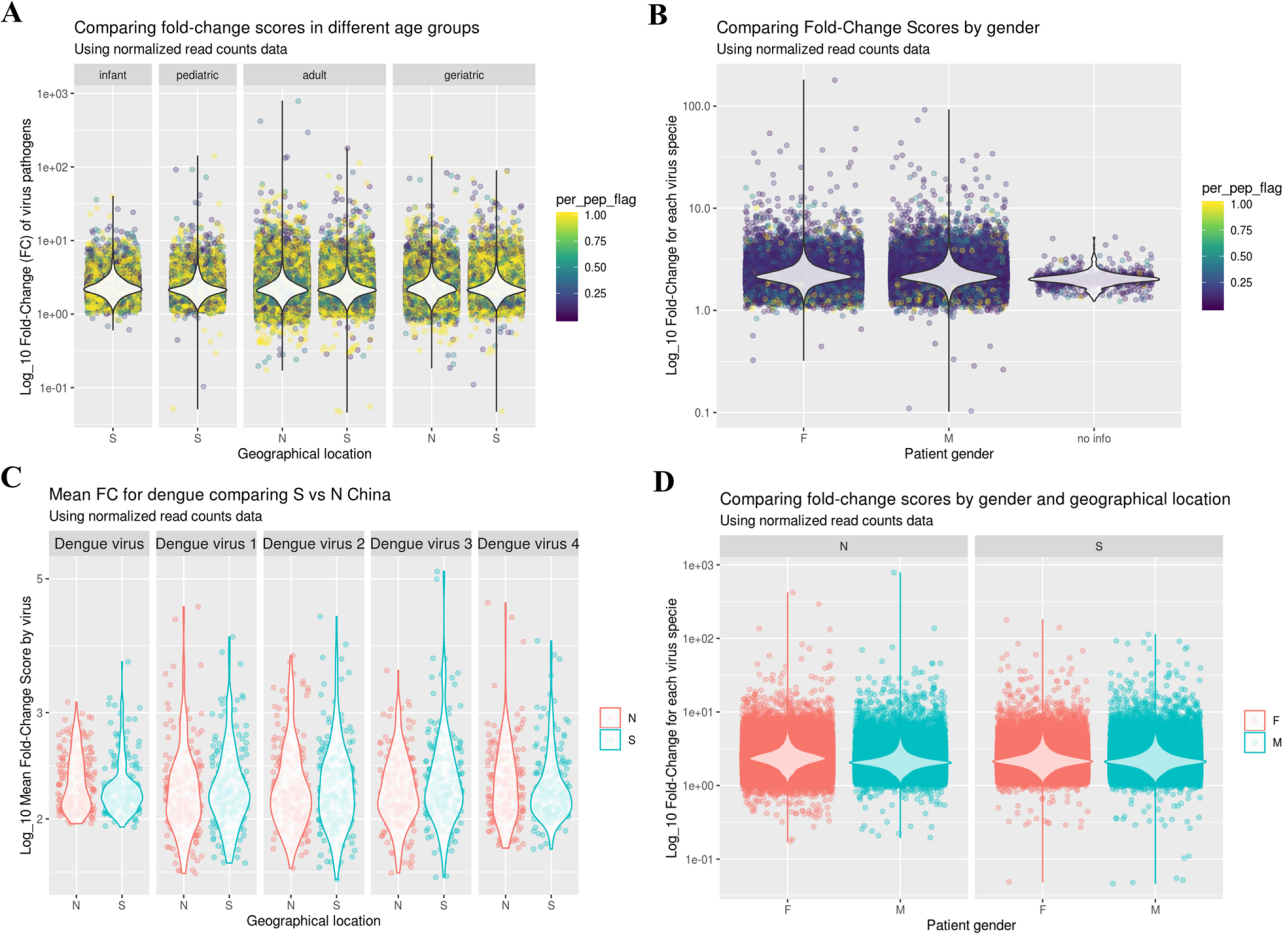
Comparison of overall immune response (FC scores) for virus by age, gender, and geographical location. **A** Comparison of FC values for virus pathogens across different age groups: infant (0 < age < 3), pediatric (3 ≤ age < 16), adult (16 ≤ age < 60), and geriatric (age ≥ 60), and geographical locations (Southern and Northern China). FC values are shown on a log scale. **B** Comparison of virus species FC scores by patient gender (female, male, and no information). **C** Mean FC for dengue virus strains (dengue virus 1–4) comparing Southern (S) and Northern (N) China. **D** Comparison of virus species FC scores by gender and geographical location (Northern and Southern China). FC scores are plotted for male (M) and female (F) patients in both regions. FC fold change, N northern China, S southern China, F female, M male

To better capture variations in immune responses across age groups, sexes, and geographical locations, we applied a log transformation to the FC values. The Log10 FC scores for various virus pathogens across age categories (infant, pediatric, adult, and geriatric) are shown in Fig. 1A, with data further stratified by geographical location. The infant and pediatric groups were sampled exclusively in southern China due to recruitment constraints in the north. While minor regional differences in FC scores were observed in adults and geriatrics, statistical analysis revealed no significant patterns (*P* > 0.05). Given the historical prevalence of dengue virus in southern China, we focused specifically on regional antibody responses to dengue virus serotypes (Fig. 1C). Statistical analysis revealed no significant differences in dengue serotype antibody levels (*P* > 0.05). Additionally, no significant gender-based differences were observed in immune responses to viral pathogens, and no significant gender-geography interactions were detected (*P* > 0.05, Fig. 1B, D).

### Antibody reactivity across age as a continuous variable

**Fig. 2:**
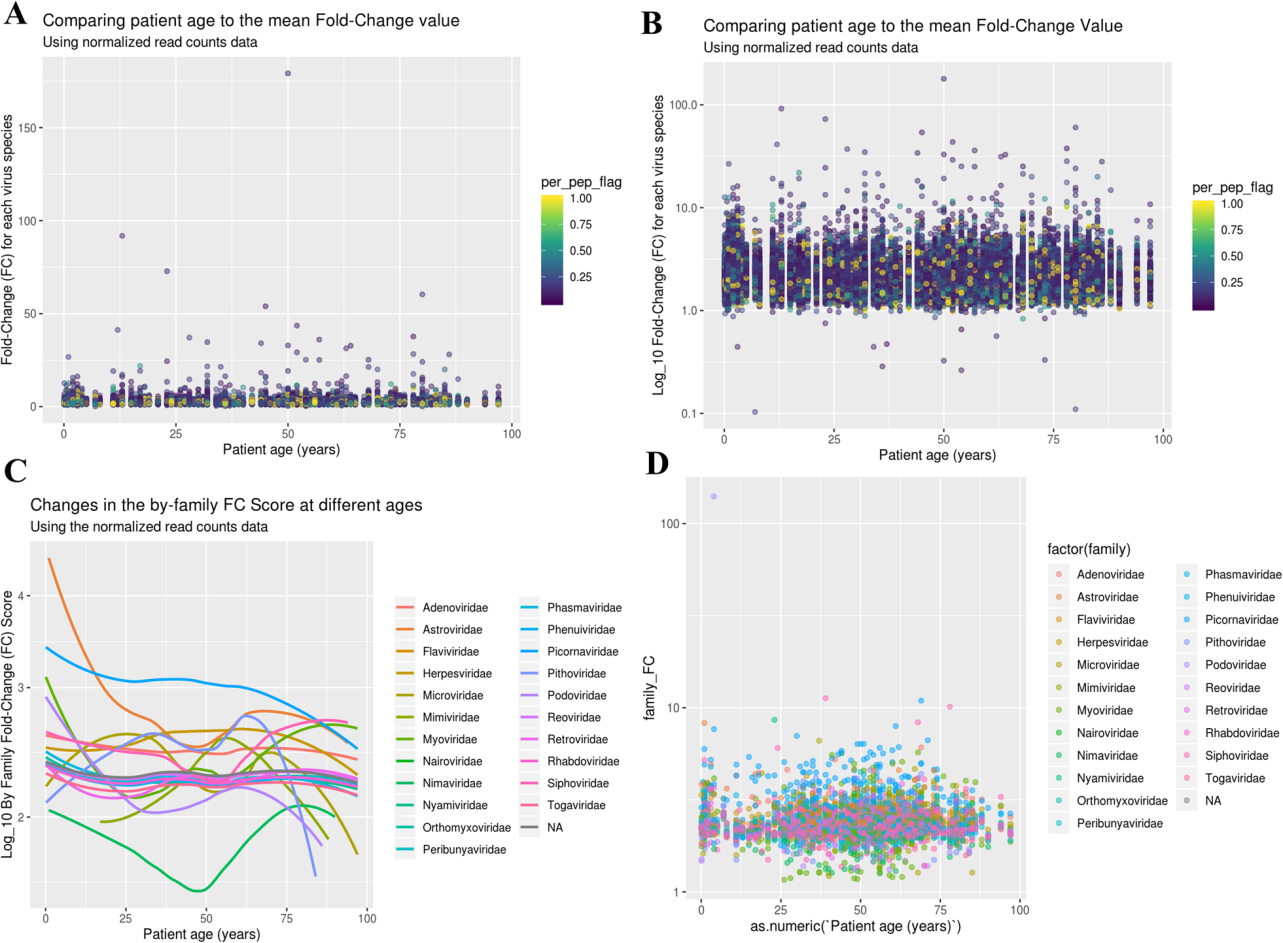
Age-related patterns in arboviral immune responses across diverse virus families. **A** Age-dependent FC responses for each virus species. **B** Log-transformed age-dependent FC responses for each virus species. **C** Age-related trends in family-specific FC scores across various arboviral families. **D** Combined scatterplot of each individual’s FC values, coded by virus family, across age. FC, fold change, per_pep_flag, per-peptide positivity score

Given the complex and potentially nonlinear nature of immune changes across the lifespan, we next assessed antibody responses as a continuous function of age (Fig. 2). In Fig. 2A and B, fold-change scores for individual virus species are plotted against participant age. Overall, the data points appear broadly distributed without clear peaks, suggesting no strong age-associated shifts in overall antibody responses. In contrast, Fig. 2C reveals more dynamic patterns when responses are aggregated by viral family. Notably, antibody responses to *Astroviridae* and *Picornaviridae* tend to decline with age, consistent with early-life exposure and durable serotype-specific immunity. Meanwhile, *Flaviviridae* responses remain relatively stable across age groups. The detailed view of individual immune responses across virus families and age groups was shown in Fig. 2D. The scattered distribution of data points across the entire age spectrum reflects high inter-individual variation, with no consistent age-related trends.

### Family-level seroreactivity stratified by age, sex, and region

**Fig. 3:**
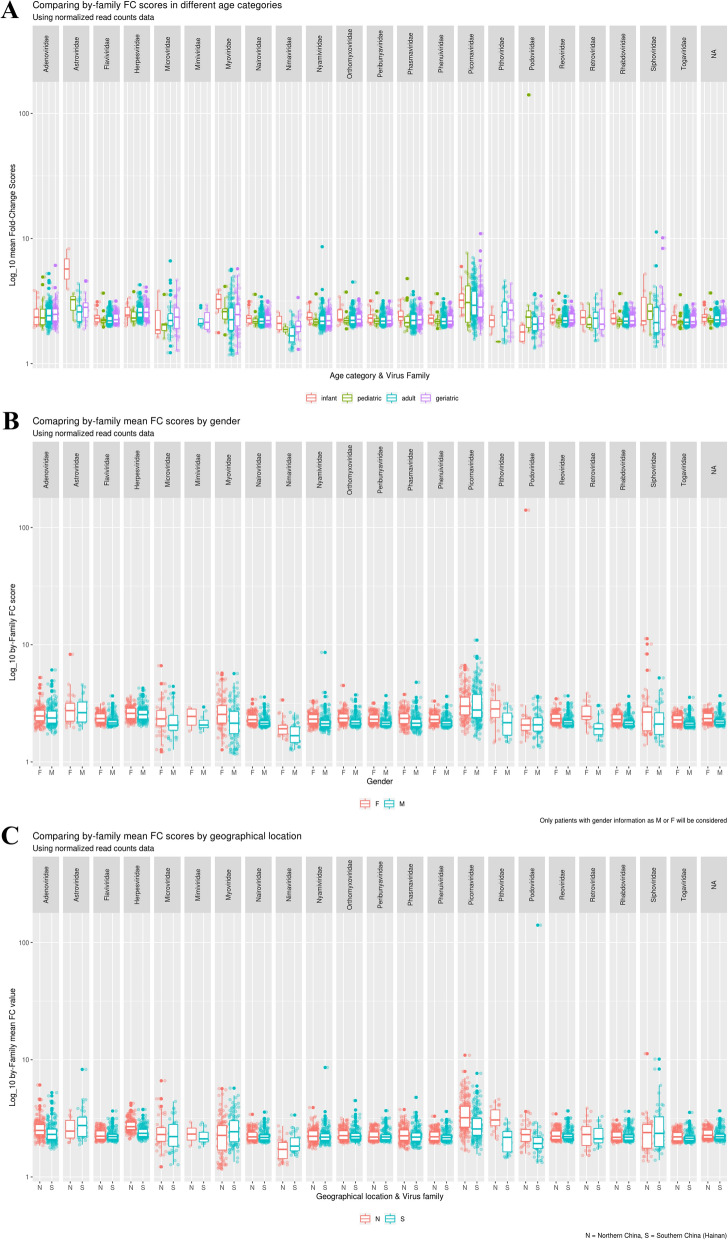
Comparative analysis of mean FC scores by virus family across age, gender, and geographical location. **A** Displays the distribution of immune response FC scores across four age categories (infant, pediatric, adult, geriatric) for various virus families. **B** Compares the mean FC scores by gender (male and female) across different virus families. **C** Examines the mean FC scores by virus family across geographical locations (North and South). FC fold change, F female, M male, N Northern China, S Southern China

**Fig. 4:**
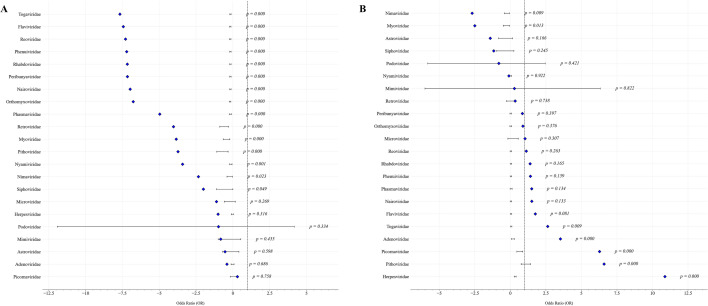
Forest plots of independent *t*-tests comparing by-family FC scores by gender and geographical location. **A** Forest plot showing the results of independent *t*-tests comparing by-family FC scores across gender (Male vs Female). **B** Forest plot showing the results of independent *t*-tests comparing by-family FC scores across geographical locations (Northern vs Southern China)

To investigate variations in arboviral immune responses, we analyzed normalized FC scores of antibody reactivity across multiple dimensions, including age, gender, and geographical location. Age-stratified analysis (infant, pediatric, adult, and geriatric; Fig. 3A) showed no statistically significant differences in immune responses to most arboviral families, including *Flaviviridae*, *Togaviridae* and *Phenuiviridae* (*P* > 0.05, Table 2). In contrast, several non-arboviral control families showed significant age-dependent differences, with *Astroviridae* (*P* < 0.001), *Podoviridae* (*P* < 0.01), and *Nimaviridae* (*P* < 0.01), displaying higher responses in younger participants. While overall immune responses were generally similar between genders, some arboviral families, notably *Togaviridae* and *Flaviviridae*, exhibited significant differences in FC values, with females showing higher FC values compared to males (*P* < 0.001, Figs. 3B, 4A). Regional analysis of arboviral families revealed notable differences between Northern and Southern China, with odds ratio analysis indicated a significantly higher likelihood of seroreactivity to *Togaviridae* in Southern China (*P* = 0.01, Figs. 3C, 4B).

**Table 2:** Results of one-way ANOVA testing for differences in FC among age groups, by virus family

Family	F	*P*
*Astroviridae*^†^	11.5938150	0.0000078
*Podoviridae*^†^	5.6720404	0.0012037
*Nimaviridae*^†^	4.7613543	0.0045112
*Myoviridae*^†^	2.8677142	0.0373892
*Phenuiviridae*	2.2202852	0.0852862
*Phasmaviridae*^†^	2.1230620	0.0967678
*Orthomyxoviridae*	2.0961115	0.1002063
*Nairoviridae*	1.9617400	0.1191827
*Rhabdoviridae*	1.8760651	0.1330350
*Reoviridae*	1.7604793	0.1541769
*Peribunyaviridae*	1.7130728	0.1637407
*Flaviviridae*	1.6876461	0.1690991
*Herpesviridae*^†^	1.5901316	0.1912231
*Togaviridae*	1.3748661	0.2499947
*Pithoviridae*^†^	1.0573003	0.3754285
*Picornaviridae*^†^	0.8011570	0.4937805
*Siphoviridae*^†^	0.6355196	0.5939393
*Nyamiviridae*^†^	0.5473525	0.6501913
*Adenoviridae*^†^	0.3331919	0.8013548
*Microviridae*^†^	0.1152617	0.9509363
*Retroviridae*^†^	0.0744737	0.9733870
*Mimiviridae*^†^	0.0003883	0.9845777

For each virus family, the F statistic and *P*-value are shown

^†^non-arboviruses included for completeness and excluded from arboviral analyses (e.g., bacteriophages or human/animal viruses that are not vector-borne)

## Discussion

This study provides the first population-based serological profiling of arboviral immunity in China using the ArboScan platform. We identified distinct regional and sex-based differences in antibody responses, while age-related patterns were largely absent. These findings highlight how demographic and ecological factors jointly shape arboviral exposure patterns, with important implications for targeted surveillance, vaccine planning, and vector control. In particular, the lack of significant differences in dengue serotype-specific responses suggests that cross-reactive antibodies, rather than type-specific immunity, may dominate the immune landscape—posing both challenges and opportunities for public health.

At a regional level, antibody profiles displayed both ubiquitous cross-reactive signals and distinct location-specific differences. In particular, common viruses (not limited to arboviruses) elicited uniformly high antibody signals, reflecting widespread exposure and cross-reactive immunity. For example, participants in southern China exhibited elevated antibody levels against mosquito-borne viruses, consistent with higher local arbovirus exposure and antigenic cross-reactivity with endemic strains; in contrast, participants in northern regions mounted stronger responses to rhinovirus A and bluetongue virus, reflecting seasonal respiratory infections and livestock-related exposures. These divergent regional patterns align with ecological evidence that mosquito virome composition is shaped by local species and environmental factors [15]. In the southern cohort, dengue virus ranked among the top MPFC species; sampling preceded dengue activity reported in Hainan before 2019 [16]. This pattern is more consistent with cumulative exposure accrued over multiple transmission cycles, including mild or inapparent infections, rather than immunological imprinting by a single outbreak [17, 18]. The detection of dengue-specific signals in asymptomatic individuals therefore provides immunological evidence of background immunity and implies ongoing, under-ascertained transmission that routine case-based surveillance may fail to capture [19].

Extending from geography to host factors, sex and age further structure the antibody landscape, with females generally exhibiting higher fold-change responses across arboviral families. This disparity may reflect enhanced interferon signaling from X-linked immune genes such as TLR7 and modulation by sex hormones [20–22]. Likewise, stratifying the data by age revealed virus-specific antibody response patterns. By contrast, stratifying by age revealed virus-specific rather than uniform shifts: although immune function remodels with age (immunosenescence), our data do not show a consistent dampening of arboviral antibody responses [23]. Importantly, the absence of a strong age association for *Flaviviridae* binding in this cohort does not contradict the higher clinical severity observed in children and older adults, because clinical severity is governed by factors (heterotypic exposure timing/ADE, immunosenescence or immaturity, and comorbidities) that are distinct from the cumulative IgG binding measured here.

Methodologically, interpreting these patterns requires emphasizing that PhIP-Seq/ArboScan quantifies cumulative IgG binding to linear epitopes rather than species-resolved, severity-linked immunity. Extensive cross-reactivity within viral families (especially *Flaviviridae*) further complicates species-level attribution from binding data [24]. Our PhIP-Seq/ArboScan readout captures IgG binding to linear epitopes and is intended to quantify cumulative exposure, not to diagnose which specific flavivirus caused an individual infection. Accordingly, serology here is best used to complement case-based surveillance, particularly where illness is mild or subclinical. When species-level resolution is required for public health decisions, orthogonal confirmation is needed (paired acute-convalescent sampling with IgM and PRNT/FRNT) together with computational deconvolution of shared epitopes [25]. Prospective linkage of ArboScan signals with neutralization activity and clinical outcomes will clarify functional relevance and help identify protective epitopes for vaccine development.

Operationally, integrating multiplex serology with context-appropriate workflows is essential to transform peptide-level profiles into actionable public health intelligence. Consistent with Fischer et al. [26], reliable arboviral serology hinges on assay choice and sampling time, management of flavivirus cross-reactivity, and context-specific orthogonal workflows; multiplex approaches (including phage-display libraries) broaden surveillance but still require validation for individual-level diagnosis. Against the backdrop of silent arboviral transmission, extensive serological cross-reactivity, and limited population-level serology in Asia, ArboScan can complement routine surveillance through periodic sentinel serology and rapid, alert-triggered surveys, while longitudinal sampling and One Health data (climate, land use, vector abundance/virome, mobility) help disentangle incident infection from cumulative immunity and resolve ecological drivers. Together, these elements convert peptide-level profiles into operational intelligence for early warning, targeted vector control, and vaccine planning across diverse settings.

This study has several limitations. First, PhIP-Seq combined with the ArboScan platform primarily detects antibodies binding to linear or short continuous epitopes represented by 56-mer peptides, and may fail to capture antibodies targeting conformational or post-translationally modified epitopes, potentially underestimating the full breadth of antiviral responses. Second, our analysis focused on IgG responses, which reflect historical exposure but may miss IgM responses indicative of recent or acute infections. Third, although we observed region-specific immune reactivity patterns, we did not explicitly deconvolute antibody cross-reactivity between closely related viruses, particularly within the families *Flaviviridae* and *Togaviridae*. Future efforts using computational or competitive binding approaches will be necessary to resolve true exposure versus cross-reactive signals. Finally, while our study included diverse populations across northern and southern China, variation in sample sizes and the absence of detailed exposure histories or ecological variables may limit the generalizability of some findings.

## Conclusions

This is the first large-scale, peptide-level seroprofiling of arboviral immunity in asymptomatic populations across China using a programmable phage display platform. Our findings offer critical insights into the geographic and demographic imprinting of arboviral immune responses, with direct implications for surveillance and immunization strategies. By leveraging the ArboScan peptide library within a structured regional and demographic sampling framework, we demonstrate how seroepidemiological data can inform precision public health approaches to vector-borne disease control. This work highlights the feasibility and value of integrating broad-spectrum serology into arboviral risk assessment, particularly in the context of ecological change and underrecognized exposure.

## Data Availability

The data supporting the findings of this study are available from the corresponding author, Qianfeng Xia, upon reasonable request.

## Abbreviations

ANOVA: Analysis of variance
DNA: Deoxyribonucleic acid
F: Female
FC: Fold change
IQR: Interquartile range
IgG: Immunoglobulin G
IgM: Immunoglobulin M
M: Male
MPFC: Mean product fold change
MPPF: Mean per peptide flag
N: Northern China
PCR: Polymerase chain reaction
PhIP-Seq: Phage ImmunoPrecipitation Sequencing
S: Southern China

## References

[1] World Health Organization. Vector-borne diseases. 2024. https://www.who.int/news-room/fact-sheets/detail/vector-borne-diseases. Accessed 27 Nov 2025.

[2] Troppens D, Neyts J. Challenges in combating arboviral infections. Nat Commun. 2024;15(1):3350. 38637542 10.1038/s41467-024-47161-3 PMC11026410

[3] Utarini A, Indriani C, Ahmad RA, Tantowijoyo W, Arguni E, Ansari MR, et al. Efficacy of *Wolbachia*-infected mosquito deployments for the control of dengue. N Engl J Med. 2021;384(23):2177–86. 34107180 10.1056/NEJMoa2030243 PMC8103655

[4] Girard M, Nelson CB, Picot V, Gubler DJ. Arboviruses: a global public health threat. Vaccine. 2020;38(24):3989–94. 32336601 10.1016/j.vaccine.2020.04.011 PMC7180381

[5] Pierson TC, Diamond MS. The continued threat of emerging flaviviruses. Nat Microbiol. 2020;5(6):796–812. 32367055 10.1038/s41564-020-0714-0 PMC7696730

[6] Liang G, Li X, Gao X, Fu S, Wang H, Li M, et al. Arboviruses and their related infections in China: a comprehensive field and laboratory investigation over the last 3 decades. Rev Med Virol. 2018;28(1):e1959. 10.1002/rmv.1959 29210509

[7] Jalloh MA, Artika IM, Dewi YP, Syafruddin D, Idris I, Bernadus JB, et al. Seroprevalence of chikungunya in an asymptomatic adult population in North and South Sulawesi, Indonesia. Am J Trop Med Hyg. 2023;108(2):359–62. 36535254 10.4269/ajtmh.22-0328 PMC9896315

[8] Dellagi K, Salez N, Maquart M, Larrieu S, Yssouf A, Silaï R, et al. Serological evidence of contrasted exposure to arboviral infections between islands of the Union of Comoros (Indian Ocean). PLoS Negl Trop Dis. 2016;10(12):e0004840. 27977670 10.1371/journal.pntd.0004840 PMC5157944

[9] Gupta H, Barde PV, Singh MP, Bharti PK, Nitika N. A comprehensive overview of the burden, prevention, and therapeutic aspects of arboviral diseases in India. Commun Med. 2025;5(1):254. 40596648 10.1038/s43856-025-00968-7 PMC12214824

[10] Larman HB, Laserson U, Querol L, Verhaeghen K, Solimini NL, Xu GJ, et al. PhIP-Seq characterization of autoantibodies from patients with multiple sclerosis, type 1 diabetes and rheumatoid arthritis. J Autoimmun. 2013;43:1–9. 23497938 10.1016/j.jaut.2013.01.013 PMC3677742

[11] Morgenlander WR, Chia WN, Parra B, Monaco DR, Ragan I, Pardo CA, et al. Precision arbovirus serology with a pan-arbovirus peptidome. Nat Commun. 2024;15(1):5833. 38992033 10.1038/s41467-024-49461-0 PMC11239951

[12] Xu GJ, Kula T, Xu Q, Li MZ, Vernon SD, Ndung’u T, et al. Comprehensive serological profiling of human populations using a synthetic human virome. Science. 2015;348(6239):aaa0698. 26045439 10.1126/science.aaa0698 PMC4844011

[13] Yue Y, Liu X, Ren D, Wu H, Liu Q. Spatial dynamics of dengue fever in mainland China, 2019. Int J Environ Res Public Health. 2021;18(6):2855. 33799640 10.3390/ijerph18062855 PMC7999437

[14] Mohan D, Wansley DL, Sie BM, Noon MS, Baer AN, Laserson U, et al. PhIP-seq characterization of serum antibodies using oligonucleotide-encoded peptidomes. Nat Protoc. 2018;13(9):1958–78. 30190553 10.1038/s41596-018-0025-6 PMC6568263

[15] Liu Q, Cui F, Liu X, Fu Y, Fang W, Kang X, et al. Association of virome dynamics with mosquito species and environmental factors. Microbiome. 2023;11(1):101. 37158937 10.1186/s40168-023-01556-4 PMC10165777

[16] Liu L, Wu T, Liu B, Nelly RMJ, Fu Y, Kang X, et al. The origin and molecular epidemiology of dengue fever in Hainan Province, China, 2019. Front Microbiol. 2021;12:657966. 33841385 10.3389/fmicb.2021.657966 PMC8025777

[17] Jing Q, Li Y, Liu J, Jiang L, Chen Z, Su W, et al. Dengue underestimation in Guangzhou, China: evidence of seroprevalence in communities with no reported cases before a large outbreak in 2014. Open Forum Infect Dis. 2019;6(7):ofz256. 31304186 10.1093/ofid/ofz256 PMC6612883

[18] Bhatt S, Gething PW, Brady OJ, Messina JP, Farlow AW, Moyes CL, et al. The global distribution and burden of dengue. Nature. 2013;496(7446):504–7. 23563266 10.1038/nature12060 PMC3651993

[19] Duong V, Lambrechts L, Paul RE, Ly S, Lay RS, Long KC, et al. Asymptomatic humans transmit dengue virus to mosquitoes. Proc Natl Acad Sci USA. 2015;112(47):14688–93. 26553981 10.1073/pnas.1508114112 PMC4664300

[20] Souyris M, Cenac C, Azar P, Daviaud D, Canivet A, Grunenwald S, et al. TLR7 escapes X chromosome inactivation in immune cells. Sci Immunol. 2018;3(19):eaap8855. 29374079 10.1126/sciimmunol.aap8855

[21] Hoffmann JP, Liu JA, Seddu K, Klein SL. Sex hormone signaling and regulation of immune function. Immunity. 2023;56(11):2472–91. 37967530 10.1016/j.immuni.2023.10.008

[22] Anker M, Arima Y. Male-female differences in the number of reported incident dengue fever cases in six Asian countries. Western Pac Surveill Response J. 2011;2(2):17–23. 23908884 10.5365/WPSAR.2011.2.1.002 PMC3730962

[23] Liu W, Xu J, Pu Q, Lan M, Zhang X, Gu Y, et al. The reference ranges and characteristics of lymphocyte parameters and the correlation between lymphocyte parameters and routine health indicators in adults from China. Immun Ageing. 2022;19(1):42. 36167546 10.1186/s12979-022-00298-5 PMC9513899

[24] Madere FS, da Silva AVA, Okeze E, Tilley E, Grinev A, Konduru K, et al. *Flavivirus* infections and diagnostic challenges for dengue, West Nile and Zika viruses. Npj Viruses. 2025;3(1):36.

[25] Monaco DR, Kottapalli SV, Breitwieser FP, Anderson DE, Wijaya L, Tan K, et al. Deconvoluting virome-wide antibody epitope reactivity profiles. eBioMedicine. 2022;75:103747. 34922324 10.1016/j.ebiom.2021.103747 PMC8688874

[26] Fischer C, Jo WK, Haage V, Moreira-Soto A, de Oliveira Filho EF, Drexler JF. Challenges towards serologic diagnostics of emerging arboviruses. Clin Microbiol Infect. 2021;27(9):1221–9. 34111589 10.1016/j.cmi.2021.05.047

